# 

**Published:** 1855-10

**Authors:** 


### CONTENTS—ORIGINAL COMMUNICATIONS.

Lectures on the Duties and Proprieties of the Dental Practitioner. By Professor E. Townsend, D.D.S. Philadelphia ... 1
Caries of the Teeth. By J. Taft, D.D.S ... 34
Treatment of Dental Caries when complicated with an irritable or exposed Pulp. By James Taylor ... 42
Chlorine. (CL.—35.42. By G. Watt, D.D.S., M, D., Xenia, Ohio, ... 50
Pr. J. M. Locke's Report on his Analysis of Delabarre's, Drs. Allen and Hunter's Formulas for Continuous Gum Work. ... 59
The Dental Obturator. By A. S. Talbert, D.D.S., Lexington, Ky ... 70
Drying Cavities Peparatory to Filling ... 72
Slayton on Gutta Percha., ... 73

### PROCEEDINGS OF SOCIETIES.

American Society of Dental Surgeons, and American Dental Convention ... 77
Selected Articles ... 104
Editorial ... 110

